# Case Ascertainment in Pediatric Heart Failure Using International Classification of Disease Clinical Modification (ICD-CM) Codes

**DOI:** 10.21203/rs.3.rs-4941771/v1

**Published:** 2024-10-15

**Authors:** Lindsay J. May, Josef Stehlik, Jacob Wilkes, Zhining Ou, Nelangi M. Pinto, Antonio G. Cabrera, Martin Tristani-Firouzi, Heather T. Keenan

**Affiliations:** University of Utah; University of Utah School of Medicine; Intermountain Health; University of Utah; University of Washington; Ohio State Unviersity College of Medicine; University of Utah; University of Utah

**Keywords:** Pediatrics, heart failure, ICD code, epidemiology

## Abstract

**Background:**

Most epidemiological studies in pediatric heart failure (HF) use administrative database sources, defining patient cohorts by presence of a single HF ICD code. However, the ability of ICD codes to identify true HF patients is unknown in pediatrics. Here we describe the accuracy of HF ICD-10-CM code search algorithms, in identifying pediatric patients with HF from electronic data sources.

**Methods:**

Based on the adult HF literature, search algorithms were designed to incorporate HF ICD codes, imaging, and medications. Sensitivity, specificity, positive and negative predictive value and accuracy of the algorithms were tested among children in an advanced HF clinic (“Clinic cohort”). Top-performing algorithms were then tested in a large-scale regional electronic data warehouse (EDW), 01/2017 to 01/2020, generating the “EDW Cohort”. False positive cases were identified and characterized by chart review.

**Results:**

Within the Clinic Cohort, 78/378 patients (21%) had gold standard HF diagnoses. A search algorithm with one HF ICD coded visit was more sensitive but less specific than >1 HF ICD coded visit, (sensitivity 94% and specificity 89% versus 69% and 97%, respectively). Correspondingly, >1 ICD coded visit had a higher PPV than one ICD coded visit; 84% vs. 69%. Accuracy was similar (90% vs 91%). Presence of 1 HF ICD code combined with HF medication had high sensitivity, specificity, PPV, NPV and accuracy, all higher than the single ICD code algorithm. The “1 HF coded visit + any medication” algorithm resulted in highest accuracy (93%). Top-performing algorithms were tested in the EDW: the algorithm with > 1 HF ICD coded visit, and the algorithm with one HF ICD coded visit combined with HF medication. In the EDW Cohort, 133/248 (53.6%) patients had gold standard HF diagnoses though 115/248 (46.3%) were false positive cases; 41% of those had pulmonary over-circulation from congenital heart disease. Excluding children < 30 days old and those with a history of an isolated VSD repair, complete AVSD repair, or PDA closure further reduced the proportion of false positives to 50/248 (20%).

**Conclusions:**

A search algorithm using a single HF ICD code can have acceptable sensitivity, specificity, PPV, NPV and accuracy in identifying children with HF from within electronic medical records. Similar to adult HF literature, specificity improves by including HF medication. When applied to large data sources, however, the search algorithms result in a high proportion of patients with pulmonary overcirculation related to congenital heart disease. To narrow the population to those with myocardial dysfunction, case identification may require use of ICD codes with linked of administrative, surgical, and/or imaging databases.

## Introduction

Pediatric heart failure (HF) research and epidemiology are constrained by difficulties with case identification. To identify children with HF, investigators typically query large administrative databases, using heart failure ICD (International Classification of Disease) codes to identify a study cohort. The use of administrative data can be important in pediatric HF because this allows the creation of large cohorts to study this relatively uncommon disease; however, the sensitivity and specificity of the ICD codes used to ascertain HF cohorts have not been defined.

Inaccuracies in the identification of HF from administrative databases have been well-described in the adult HF literature. The use of a single HF ICD code to identify “true”, adjudicated HF among adults in large administrative databases has poor sensitivity and specificity.[1, 2] Fortunately, performance of ICD codes to identify HF can be improved by using search algorithms that combine > 1 HF ICD code with supplemental imaging and medication data.[3–5] Algorithms can be tailored to provide a variety of potential approaches to HF patient identification. For example, search algorithms with high positive predictive value would be well-suited to identify a patient cohort for clinical trial research, whereas algorithms with high sensitivity may be most useful for epidemiologic research purposes.

An algorithm-based approach to patient identification does not currently exist in the pediatric HF literature. To build cohorts of pediatric HF patients, there is a need for well-characterized, ICD code-based search algorithms that can be used in large data sources, until pediatric HF registries are well-established.

The aim of this study is to describe the sensitivity, specificity, positive and negative predictive values (PPV and NPV, respectively) of eight search algorithms using HF ICD-CM codes and HF medication codes within electronic data sources, to identify pediatric patients with adjudicated HF. Given the high number of false positives reported in the adult HF and pediatric congenital heart disease literature when using ICD-code based search algorithms,[1–5, 14] we also aimed to report the proportion of false positive HF patients identified by using ICD-CM code search algorithms in a large administrative data source. Findings of this study will inform case ascertainment in future work using large health care systems and national databases to accurately describe the epidemiology and long-term outcomes of children with HF.

## Materials and methods

### Overview

Initial algorithms were developed based on the adult HF literature, using HF ICD-CM codes and medication data. We then identified a subgroup of children (the Clinic Cohort), cared for in the Intermountain advanced HF clinic, and reviewed their medical records to evaluate whether their notes stated a current or prior HF diagnosis. Those with current or prior HF explicitely stated in the notes were considered adjudicated “gold standard” HF patients, whereas those without this documentation were considered not to be adjudicated HF patients. Next, algorithm performance was evaluated in this Clinic Cohort to calculate sensitivity, specificity, PPV, NPV and accuracy. We then evaluated the top-performing algorithms in the Intermountain Health large-scale regional electronic data warehouse (the EDW Cohort). The number and type of false positive cases was reported.

### Search Algorithms

Eight search algorithms were developed (Table I, “Algorithm description”) using HF ICD-CM codes as the central component, since ICD codes are widely available in large electronic datasets. The HF ICD-9 and ICD-10 CM codes are shown in Supplementary Table I; in 2015, codes transitioned to the ICD-10 system. Algorithm components included the number of HF ICD-CM codes assigned to the patient, HF medication records, and presence of an echocardiogram CPT (Current Procedural Terminology^®^) code within 3 months of the clinical encounter during which a HF code was assigned. Because most administrative data sources in pediatrics focus on inpatient data, we collected inpatient medication data (from inpatient medication administration records) for use in the algorithms. Medications included enalapril, lisinopril, captopril, losartan, digoxin, carvedilol, metoprolol, milrinone, dobutamine, and spironolactone.[6] An algorithm was built with and without spironolactone since it may be a less specific medical therapy for HF in children.

Gold standard HF diagnosis: To adjudicate the Clinic Cohort patients’ HF diagnosis (“heart failure” or “no heart failure”), the most recent HF clinic note within the study timeframe 01/2017 and 12/2020 was retrospectively reviewed. Patients were given a gold standard diagnosis of HF if: (1) the clinician who authored the note explicitly stated that the patient had HF at the time of the encounter or had a history of HF, or (2) if the note explicitly stated a New York Heart Association/Ross (NYHA) Class II-IV HF or an American College of Cardiology/American Heart Association (ACC) HF Stage C or D. [7–9] These standard HF classifications are widely used in adult HF and are also applied to pediatric patients with myocardial dysfunction (systolic, diastolic, or both) to provide a semi-quantitative measure of their HF status (Supplementary Table II). Of note, in this centralized HF clinic, notes are standardized to include the NYHA Class and ACC Stage.

By using this gold standard diagnosis, we aimed to specifically exclude patients with “heart failure” in the setting of simple left to right shunt congenital heart disease lesions without myocardial dysfunction. Heart failure is broadly defined as “…a clinical and pathophysiologic syndrome resulting from ventricular dysfunction, volume, or pressure overload, alone or in combination”.[9, 10] As such, children with pulmonary over-circulation from simple congenital heart disease (CHD) lesions such as a patient ductus arteriosus or a large ventricular septal defect can sometimes be classified as having a form of HF. However, children with these correctable shunt lesions and intact myocardial function have distinctly better outcomes than children with HF due to myocardial dysfunction, thus are not the target population for pediatric HF epidemiologic study. Therefore, it would be beneficial for search algorithms to identify/exclude patients with simple correctable left to right shunt lesions. With this in mind, we chose to focus our gold standard diagnosis on children with HF characterized by myocardial dysfunction-NYHA/Ross Class and ACC/AHA Stages are not used in the contemporary era to describe patients with pulmonary over-circulation in the absence of myocardial dysfunction.

### Identification of the Clinic Cohort:

The Clinic Cohort was comprised of 378 patients, ≤18 years old, who received care in the Intermountain advanced pediatric heart failure clinic between 01/2017 and 12/2020 (Figure I). This time frame was chosen to reflect a contemporary cohort and a single electronic medical record. This clinic provides care for children receiving active HF management and also children referred for HF screening based on risk factors such as underlying diagnosis and family history. This clinic was chosen as the source population because this population is enriched with “true” HF patients, thus medical record review was feasible; an a priori estimate of the prevalence of adjudicated HF among patients in this clinic was ~ 20%, therefore, a sample size of 357 patient records for the Clinic Cohort was required to calculate sensitivity and specificity of the algorithms.

Hospital readmission is very common among children with HF,[11] so we anticipated that a hospitalization identifier in the algorithms may improve specificity. As a result, we identified which patients in the Clinic Cohort had and had not been hospitalized during the study time frame. All ICD-CM codes for the clinic visit, and codes for inpatient HF medications were also abstracted for each patient.

### Algorithm application to the Clinic Cohort:

Search algorithms in Table I were applied to the Clinic Cohort. We calculated the algorithms’ sensitivity, specificity, PPV, NPV, and accuracy against the gold standard, which was implemented by“R” version 4.1.2.[12]

### Larger-scale algorithm testing in the EDW Cohort:

Based on the sensitivity, specificity, PPV, NPV and accuracy, the two top-performing algorithms were chosen for testing in the larger Electronic Data Warehouse (EDW). The Intermountain Health (IH) EDW contains inpatient and outpatient HF records for children across 6 states, at any IH facility. The EDW allows easy access to demographic information, medications, billing, and coding data that can be queried for all children seen with the entire IH system. The EDW is linked to electronic medical records, enabling chart review for confirmation of diagnoses, procedures, and validation of cases. The EDW is thus a rich information source for algorithm testing.

In combination, the two top-performing search algorithms were applied to the EDW during the time frame 01/2017 and 01/2020, generating the “EDW Cohort”. If the algorithms performed as expected, the EDW Cohort would include a high proportion of children with HF with relatively few false positives. To generate the EDW Cohort, patients ≤18 years old were incuded. To replicate a search strategy used in large administrative databases (such as PHIS, Pediatric Health Information System^®^, or the KID database) which are inpatient-focused, we identified which patients had or had not been hospitalized during the study time frame. For testing in the EDW, we excluded patients who did not have at least one CPT code for an echocardiogram in the system, as these patients would not likely have true HF.

Linked medical records for the EDW cohort were reviewed using methods above to adjudicate the HF diagnoses (using the gold standard definition), thereby evaluating the efficacy of the search algorithms in this large data source. To better understand the limitations of the search algorithms, number and characteristics of the false positive HF patients were reviewed in detail including linked cardiac catheterization and surgical data to confirm the underlying diagnoses.

## Results

### Search algorithms and gold standard diagnoses in the Clinic Cohort

Algorithms in Table I and gold standard criteria for a HF diagnosis were applied to the Clinic Cohort (N = 378). The Cohort was subdivided into two groups as shown in Figure I: those who were or were not hospitalized during the study period [the “hospitalized subgroup” N = 134/378 (35%) and the “outpatient subgroup” N = 244/378 (65%), respectively]. Within the Cohort, 78/378 patients (21%) had a gold standard diagnosis of HF: 65/134 (48.5%) in the hospitalized subgroup and 13/244 (5.3%) in the outpatient subgroup.

Performance of each search algorithm is shown in Table I with confusion matrices shown in Supplementary Table IIIa. Presence or absence of an echocardiogram CPT code did not change the composition of the Cohort, as all patients had received an echocardiogram. Algorithm (Alg) A and Alg C included a single HF ICD-CM coded visit (Table I), whereas Alg B and Alg D included >1 HF ICD-CM coded visit. These four algorithms were applied to the overall Clinic Cohort and the hospitalized subgroup, respectively. In the overall Clinic Cohort, Alg A (the criteria of one HF ICD coded visit) was more sensitive but less specific than >1 HF ICD coded visit (Alg B), with sensitivity of 94% and specificity 89% compared with 69% and 97%, respectively (Table I). Correspondingly, Alg B (>1 HF ICD coded visit) had a higher PPV than the use of Alg A (one HF ICD coded visit); 84% compared with 69%. The accuracy of the two methods was similar (90% vs 91%).

### Clinic Cohort: hospitalized subgroup

Among the “hospitalized subgroup” Alg C-J were applied (Table I, Alg C-J and Supplementary Table IIIb). As in the overall Clinic Cohort, Alg D (>1 HF ICD-CM coded visit in the hospitalized subgroup) improved specificity and PPV at the expense of sensitivity when compared to a single HF ICD coded visit algorithm (Alg C).

The presence of 1 HF ICD coded visit in combination with any HF medication (Alg E) resulted in high sensitivity, specificity, PPV, NPV and accuracy, all of which were higher than the single HF ICD coded visit algorithm (Alg C). Of note, the “1 HF coded visit + any medication” algorithm (Alg E) resulted in the highest accuracy (93%). Performance parameters changed very little by excluding spironolactone (Alg F).

When limiting the criteria to include only specific medications (Alg G-J), algorithm sensitivity declined. For example, the highest specificity (99%) was seen among algorithms with >1 HF ICD coded visit (Alg D) and 1 HF ICD coded visit with carvedilol during an inpatient encounter (Alg G), though the sensitivities of these two algorithms were among the lowest (48% and 32%, respectively).

### Search algorithms in the EDW

The two top-performing algorithms from the inpatient group were applied to the Intermountain EDW: the algorithm with > 1 HF ICD coded visit (Table I, Alg D; a highly specific algorithm), and the algorithm with one HF ICD coded visit in combination with any medication (Table I, Alg E; an algorithm with high accuracy and good balance of sensitivity/specificity and PPV/NPV). The “EDW Cohort” of 248 patients was identified by applying these two algorithms during the time frame 01/2017 and 01/2020.

For this EDW Cohort, 133/248 (53.6%) patients were found to have a gold standard diagnosis of HF, whereas 115 patients (46.3%) were false positive cases. Based on medical record review, 47/115 (41%) of these false positive cases were children with pulmonary over-circulation from congenital heart disease lesions including atrioventricular septal defects, ventricular septal defects, and patent ductus arteriosus. Another 22/115 (19%) false positive cases were neonates without HF.

Excluding children under 30 days of age from the search algorithm removed 16 false positive patients, thus reducing the proportion of false positives from 115/248 (46%) to 99/248 (40%). Linked medical records, cardiac catheterization CPT codes, and institutional surgical data (Society of Thoracic Surgeons primary procedure codes) were used to refine these remaining 99 false positive HF patients. Removal of those with isolated VSD repair or complete AVSD repair without other surgical procedures, or PDA closure (transcatheter or surgical) further reduced the proportion of false positive patients to 50/248 (20%).

## Discussion

In this study we illustrate that a search algorithm using a single HF ICD-CM code can have acceptable sensitivity, specificity, PPV, NPV and accuracy in identifying children with HF from within electronic medical records, but not surprisingly, algorithm performance dependends on the data source. The specificity of this search algorithm is improved by including HF medication as an additional criterion. These findings are generally similar to the contemporary adult HF literature,[3] in which search strategies using more than 1 HF ICD coded visit and/or medications further improve the PPV of the algorithms at the expense of the sensitivity. Of note, in our cohort, an inpatient admission was also associated with a higher likelihood of having an adjudicated HF diagnosis.

Despite the initially good test characteristics of the algorithms in the Clinic Cohort (a population with a high prevalence of HF), we captured many false positive patients when the algorithms were used in a larger data source with a lower prevalence of HF- the Intermountain Electronic Data Warehouse which closely mirrors the Pediatric Health Information System (PHIS) data source. Based on our findings, up to 46% of patients captured were false positives. A high number of these patients received HF ICD codes in the setting of simple congenital heart disease consisting of left to right shunts without myocardial dysfunction. This suggests that when using administrative data sources for HF population-based research, search algorithms may still include a high proportion of patients without myocardial dysfunction. While this specific concern is unique to pediatric HF, there are numerous other reasons for inconsistent or inaccurate ICD code assignment in large pediatric cohorts, such as limitations in medical documentation, variable coding practices, misspecification, and upcoding.[13] These issues also exist in the congenital heart disease literature, in which ICD code search strategies include a high proportion of false positives (as high as 69.1%) necessitating ICD code combinations in tailored search algorithms. [14] With this context, it is clear that a nuanced approach to case ascertainment is required for pediatric HF research.

Authors in the pediatric HF literature commonly cite ICD code challenges as a methodologic limitation[15–17] because until now, the accuracy of a single ICD code approach has not been reported. This concern is evident in recent work using PHIS to describe pediatric HF epidemiology. In this study, >50% of children identified as having HF (based on ICD codes) with congenital heart disease had left to right shunts such as ventricular septal defects, or patent ductus arteriosus.[14] While it is possible that these children had true myocardial dysfunction and HF in addition to simple congenital heart disease, it seems more likely that these children received a HF ICD code in the setting of pulmonary over-circulation. Because these patients clearly have a surgically correctable cause of “heart failure” many HF investigators would prefer to exclude them from HF study cohorts, because their outcomes will differ substantially from those with myocardial dysfunction. Based on our findings, the application of an ICD-code search algorithm followed by use of institutional surgical data, or linked data sets such as the Society of Thoracic Surgeons (STS) National Database, can ensure a higher proportion of patients with adjudicated HF from a large data source. Application of STS Primary Procedure codes can also be used to identify children with single surgical interventions for simple shunt lesions, and removal of those patients from the HF study cohort could ensure a cleaner epidemiologic cohort of children with myocardial dysfunction and the syndrome of HF. Other options to characterize patients could include linked cardiac catheterization records or CPT codes for cardiac surgeries and procedures.

This study generates several meaningful contributions. First, the algorithms tested in this study provide an initial framework for case ascertainment in future pediatric HF research, and for the first time, we quantify the performance of ICD code search algorithms. This valuable framework serves as an important cornerstone for next steps in research. Second, we not only describe a high number of false positive HF patients in a n administrative database cohort, but we were able to identify potential means of refining a HF cohort by age or by incorporating surgical or procedural records. In general, these contributions are relevant because optimal case ascertainment using electronic datasets remains critical to improve our understanding of HF epidemiology among children at regional and national levels.

While registries that collect longitudinal pediatric HF data will be powerful future resources for epidemiologic study and may not require ICD codes for case identification, these registries are currently limited in scope.[18–20] Thus, datasets that include electronic health data such as regional datasets like the EDW, health care payor data, and multi-institutional datasets remain an important source of generalizable pediatric HF data. The ICD code-based approaches and refinements we describe in this study could be useful in identifying the population of interest from these datasets.

There are several limitations to this study. The first limitation is that for feasibility, we used a relatively small population for study. While it is possible that a larger data set may result in different algorithm performance, our findings mirror those in the adult literature, and also those described in the CHD literature when using ICD codes for case ascertainment.[14] It is also noteworthy that replicating this research with exclusively ICD-9 or ICD-10 codes may not yield the exact algorithm performance as reported in this study. Fortunately however, crosswalk systems exist to convert between ICD-9 and ICD-10 codes as needed so our work could be replicated using ICD-9 or ICD-10 codes, or both if needed based on the era of interest.

A unique strength of this study is that we were able to assign gold standard HF diagnoses using clinical chart review. Chart review also made it possible to characterize the “false positive” HF patients in the data set, allowing us to propose refinement strategies for the search algorithms. Another strength of this study is that we were able to evaluate the search algorithms in a group of patients with or without a history of hospitalization. This differs from many administrative datasets in which only inpatient data are collected.

## Conclusions

This study provides an important framework for interpreting the current HF literature as well as future approaches to case ascertainment in pediatric HF. We illustrate the value of combining HF medication records with HF ICD codes in search algorithms to improve accuracy in identifying HF patients in electronic databases. This study also highlights the challenges in identifying young infants with pulmonary over-circulation in HF datasets- this is a population distinct from the typical HF patient with myocardial dysfunction and should be studied separately. Our findings can improve the rigor of ongoing research in pediatric HF while powerful future data sources mature. Future directions will include validation of the algorithms in multi-institutional datasets, while using complimentary linked surgical data sets.

## Figures and Tables

**Figure 1:**
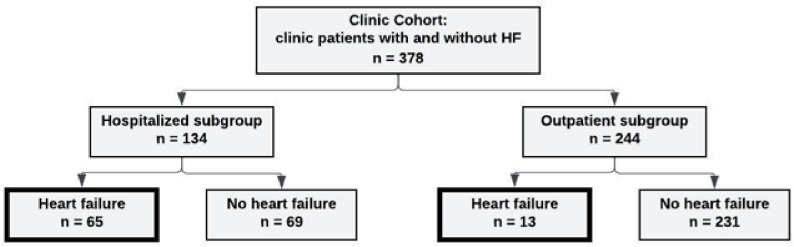
Clinic Cohort subgroups and HF ICD-10 code assignment. The Clinic Cohort includes patients receiving HF screening or management in the IH Pediatric HF Clinic between 01/2017 and 12/2020. The “Outpatient subgroup” refers to those who were not hospitalized during the study period. The “Hospitalized subgroup” refers to the subgroup of patients who had at least one hospitalization during the study period. “Heart failure” is defined as the gold standard HF diagnosis (shown in the bold rectangles), representing patients with documented current or prior HF.

**Table I.** Algorithm performance in the Clinic Cohort and hospitalized subgroup.

Alg	Algorithm description	Sensitivity	Specificity	PPV	NPV	Accuracy
	Overall Clinic Cohort					
A	1 HF ICD coded visit	94%	89%	69%	98%	90%
B	>1 HF ICD coded visit	69%	97%	84%	92%	91%
	Hospitalized subgroup					
C	1 HF ICD coded visit	92%	88%	88%	92%	90%
D	>1 HF ICD coded visit	48%	99%	97%	67%	74%
E	1 HF ICD coded visit + any medication*	94%	93%	92%	94%	93%
F	1 HF ICD coded visit + any medication* minus spironol.	91%	93%	92%	91%	92%
G	1 HF ICD coded visit + carvedilol	32%	99%	95%	61%	66%
H	1 HF ICD coded visit + milrinone	55%	96%	92%	69%	76%
I	1 HF ICD coded visit + enalapril	48%	93%	86%	65%	71%
J	1 HF ICD coded visit + carvedilol/enalapril/milrinone	77%	93%	91%	81%	85%

Alg = algorithm, PPV = positive predictive value, NPV = negative predictive value, ICD = International Classification of Disease, spironol = spironolactone

* “any medication” includes: enalapril, lisinopril, captopril, losartan, digoxin, carvedilol, metoprolol,milrinone, dobutamine, and spironolactone
